# Renal replacement therapy after cardiac surgery: do not ask “When”, ask “Why”

**DOI:** 10.1186/s13054-017-1818-7

**Published:** 2017-09-05

**Authors:** Stéphane Gaudry, David Hajage, Didier Dreyfuss

**Affiliations:** 1 AP-HP, Service de Réanimation Médico-Chirurgicale, Hôpital Louis Mourier, F-92700 Colombes, France; 2 French National Institute of Health and Medical Research (INSERM), UMR_S1155, Remodeling and Repair of Renal Tissue, Hôpital Tenon, F-75020 Paris, France; 3 AP-HP, Hôpital Pitié-Salpêtrière, Département de Biostatistiques, Santé Publique et Information Médicale, F-75013 Paris, France; 4 Université Paris Diderot, IAME, UMR 1137, Sorbonne Paris Cité, F-75018 Paris, France; 5 Present address: Intensive care unit, hôpital Louis Mourier, 178 rue des Renouillers, 92110 Colombes, France

We read with interest the article published by Zou et al. [1] regarding the timing of renal replacement therapy (RRT) initiation in patients with acute kidney injury (AKI) post-cardiac surgery. The authors claim that their meta-analysis shows that early RRT initiation decreases 28-day mortality in this context.

This meta-analysis included a mix of observational (retrospective and prospective) studies (n = 10) and randomized controlled trials (RCTs; n = 5). The observational studies had, unfortunately, a major flaw with regard to answering the question of RRT initiation. Indeed, they included only patients who actually received RRT but not patients who did not receive RRT (despite severe AKI). Remarkably, these latter are those who probably have the best prognosis [2]. That is why experts consider that comparing two strategies of RRT initiation rather than so-called early versus late RRT constitutes the only adequate study design [3, 4]. In other words, an early RRT initiation strategy in which all patients receive RRT must be compared with a delayed strategy in which some patients receive RRT because they reach pre-specified criteria and others do not receive RRT because of either renal function recovery or death.

Interestingly, the authors performed a subgroup analysis based on study design. In cohort studies, early RRT initiation was associated with significant decrease of 28-day mortality (*p* < 0.00001). In contrast this was not the case for RCTs (*p* = 0.11).

This highlights that the right question is not “when to start RRT” which underlies that all patients receive RRT (early or late) but “why start RRT”. Indeed, research in the field should now focus on the criteria which mandate RRT in different contexts, including post-cardiac surgery.

The HEROICS trial by Combes et al. (which is included in the present meta-analysis) published in 2013 is the largest RCT (with high methodological quality) on RRT initiation strategies post-cardiac surgery [5]. It shows that, for patients with post-cardiac surgery shock, early high-volume hemofiltration did not improve day 30 mortality and other patient-centered outcomes compared with a conservative strategy (delayed RRT only for persistent severe AKI). Among patients included in the conservative strategy, 36% survived without ever having started RRT.

In conclusion, pending results of ongoing RCTs (NCT02568722), we think that, to date, a conservative strategy to initiate RRT in post-cardiac surgery is an acceptable approach that will allow many patients to recover renal function without the risk of RRT.

## Abbreviations

AKI: Acute kidney injury
RCT: Randomized controlled trial
RRT: Renal replacement therapy
